# A Method for Extrinsic Parameter Calibration of Rotating Binocular Stereo Vision Using a Single Feature Point

**DOI:** 10.3390/s18113666

**Published:** 2018-10-29

**Authors:** Yue Wang, Xiangjun Wang, Zijing Wan, Jiahao Zhang

**Affiliations:** 1 State Key Laboratory of Precision Measuring Technology and Instruments, Tianjin University, Tianjin 300072, China; wy2012110040@163.com (Y.W.); zjwan@tju.edu.cn (Z.W.); q17695949504@163.com (J.Z.); 2 Key Laboratory of MOEMS of the Ministry of Education, Tianjin University, Tianjin 300072, China

**Keywords:** extrinsic parameter calibration, binocular stereo vision, single feature point

## Abstract

Nowadays, binocular stereo vision (BSV) is extensively used in real-time 3D reconstruction, which requires cameras to quickly implement self-calibration. At present, the camera parameters are typically estimated through iterative optimization. The calibration accuracy is high, but the process is time consuming. Hence, a system of BSV with rotating and non-zooming cameras is established in this study, in which the cameras can rotate horizontally and vertically. The cameras’ intrinsic parameters and initial position are estimated in advance by using Zhang’s calibration method. Only the yaw rotation angle in the horizontal direction and pitch in the vertical direction for each camera should be obtained during rotation. Therefore, we present a novel self-calibration method by using a single feature point and transform the imaging model of the pitch and yaw into a quadratic equation of the tangent value of the pitch. The closed-form solutions of the pitch and yaw can be obtained with known approximate values, which avoid the iterative convergence problem. Computer simulation and physical experiments prove the feasibility of the proposed method. Additionally, we compare the proposed method with Zhang’s method. Our experimental data indicate that the averages of the absolute errors of the Euler angles and translation vectors relative to the reference values are less than 0.21° and 6.6 mm, respectively, and the averages of the relative errors of 3D reconstruction coordinates do not exceed 4.2%.

## 1. Introduction

In recent years, with the advancement in computer vision and image technology, binocular stereo vision has been extensively used in three-dimensional (3D) reconstruction, navigation, and video surveillance. This vision method requires that the cameras possess a higher calibration accuracy and better real-time calibration to satisfy the requirements of practical engineering applications. Therefore, research on camera calibration technology of binocular stereo vision is important both theoretically and practically.

Camera calibration technology is primarily divided according to traditional [1,2,3] and self-calibration [4,5,6] methods. Traditional calibration methods require precision-machined targets, which employ the known 3D world coordinates of control points and their image coordinates to calculate the cameras’ intrinsic and extrinsic parameters. These methods include the direct linear transformation (DLT) [7], Tsai’s radial alignment constraint (RAC) [8], Wen’s iterative calibration [9], and double-plane calibration [10]. The traditional methods have high precision, but their algorithm is complex and the calibration process is time consuming and laborious. Hence, its practical application will be significantly limited. In the 1990s, Faugeras and Maybank [11] first proposed the idea of self-calibrating cameras. The self-calibration methods only require the constraint relation from the image sequence without the aid of a calibration block, possibly allowing the camera parameters to be obtained online and in real time. This method can satisfy the requirements in some special occasions where the cameras’ focal length should often be adjusted or the cameras’ position will move according to the surrounding environment.

In 1992, Faugeras proposed a self-calibration method by directly solving the Kruppa equation [12], which is computationally complex and sensitive to noise. Given the difficulty in solving the Kruppa equation, Hartley et al. proposed a QR decomposition method in 1994 by using the hierarchical step-by-step calibration [13]. The projection matrix is decomposed by QR, but the method requires the initial value for an effective calibration. Triggs et al. proposed in 1997 a camera self-calibration method based on an absolute quadratic surface [14], which is more effective than the method based on the Kruppa equation with regard to inputting multiple images. The camera self-calibration method based on an active vision system, which controls the camera to perform special motions and uses multiple images captured from various positions to calibrate the camera, is represented using the linear method according to two groups of three-direction orthogonal motions proposed by Ma in 1996 [15]. Generally, the self-calibration methods are poor robustness, and calibration accuracy is lower than that of traditional methods. However, the self-calibration method can effectively utilize various constraints unrelated to the motion of the cameras and some prior knowledge, and render the algorithm more simple and practical. Therefore, the self-calibration method is more flexible and can be utilized in a wider range of applications.

In this work, we primarily investigate the implementation of the camera self-calibration. Hitherto, researchers have performed numerous related works. The typical self-calibration algorithms are to estimate camera parameters through iterative optimization with numerous matching features. For example, Zhang proposed a local–global hybrid iterative optimization method by using the bundle adjustment algorithm and SIFT points matching relationship [16]. Wu proposed a complete model for a pan–tilt–zoom (PTZ) camera. The camera parameters can be quickly and accurately estimated using a series of simple initialization steps followed by a nonlinear optimization from ten images [17]. Junejo provided a novel optimized calibration method for cameras with pure rotation or pan–tilt rotation from two images [18]. These methods presented good calibration accuracy, but were time consuming and unable to implement online calibration. Presently, most camera self-calibration algorithms utilize various constraints in natural scenes. Echigo presented a camera calibration method by using three sets of parallel lines in which the rotation parameters were decoupled from the translation parameters [19]. Song established a calibration method for a dynamic PTZ camera overlooking a traffic scene, which automatically used a set of parallel lane markings and the lane width to compute the focal length, tilt angle, and pan angle [20]. Schoepflin presented a new three-stage algorithm to calibrate roadside traffic management cameras by estimating the lane boundaries and the vanishing point of the lines along the roadway [21]. Kim and Hong proposed a nonlinear self-calibration method of rotating and zooming cameras by using inter-image homography from refined matching lines [22]. However, these methods are suitable for the camera calibration under static conditions, which will not work if the special markers like parallel lines are missing during the rotation of the cameras. In order to solve this problem, Muñoz Rodríguez performed binocular self-calibration by means of an adaptive genetic algorithm based on a laser line [23] and presented an online self-camera orientation for mobile vision based on laser metrology and computer algorithms [24], which avoided calibrated references and physical measurements. Self-calibration algorithms were also performed well by combining with the active vision measurement method. Ji introduced a new rotation-based camera self-calibration method that requires the camera to rotate around an unknown but fixed axis twice [25]. Cai proposed a simple method to obtain the camera intrinsic parameters by observing one planar pattern in at least three different orientations [26]. These methods improved traditional self-calibration algorithm, but could not satisfy the requirements of real-time calibration.

In order to achieve fast calibration of the camera, several methods simplified the camera model. Tang proposed a non-iterative self-calibration algorithm for upgrading the projective space to the Euclidean space [27], which combined the typically used metric constraints, including zero skew and unit aspect ratio constant principal points. Yu [28] presented a self-calibration method for moving stereo cameras and introduced some reasonable assumptions, such as principle points located at the image center, camera axis perpendicular to the camera plane, and constant focal length of stereo cameras during movement, to simplify the camera model. De Agapito proposed a linear self-calibration method for a stationary but rotating camera under the minimal assumption of zero skew, known pixel aspect ratio, and known principal point [29]. Once the camera model is simplified, the number of feature points for calibration can be reduced. Sun [30] proposed a novel and effective self-calibration approach for robot vision, which both the camera intrinsic parameters and the hand-eye transformation were estimated by using only two arbitrary feature points. Chen put forward a two-point calibration method for soccer cameras in a narrow field of view, in which only two point correspondences were required given the prior knowledge of base location and orientation of a PTZ camera [31]. The above two methods are rapid and avoid the convergence problems of iterative algorithms.

In this study, we present a novel self-calibration method of BSV with rotating and non-zooming cameras by using a single feature point. The intrinsic parameters of the left and right cameras are estimated in advance by using Zhang’s method, as well as the rotation matrix and translation vector at the initial position. The left and right cameras can rotate in the horizontal and vertical directions. Thus, only the two rotation angles for each camera, denoted by pitch and yaw, must be calculated after the rotation. According to the homography of the feature point before and after the rotation, one quadratic equation for the tangent value of the pitch for each camera can be derived. The closed-form solutions of the pitch and yaw can be obtained with known approximate values of the pitch obtained using the SBG angle measuring system. Thus, the rotation angles of the left and right cameras in two directions can be calculated linearly, and then, the extrinsic parameters of the binocular stereo vision after rotation can be obtained. The proposed calibration algorithm is non-iterative and can quickly complete the extrinsic parameter calibration of the rotating cameras, rendering the possibility of estimating the 3D coordinates in real time for the dynamic stereo vision, which can be used for fast positioning of dynamic targets.

The remainder of this paper is organized as follows: Section 2 primarily describes the mathematical model of the BSV with rotating and non-zooming cameras and introduces the extrinsic parameter calibration algorithm by using a single feature point. Section 3 discusses the feasibility of the calibration method. Section 4 explains the virtual simulation and physical experiments to verify the performance of the proposed method. Finally, Section 5 elaborates the conclusions.

## 2. Principles and Methods

### 2.1. Camera model of the Binocular Stereo Vision

**Figure 1:**
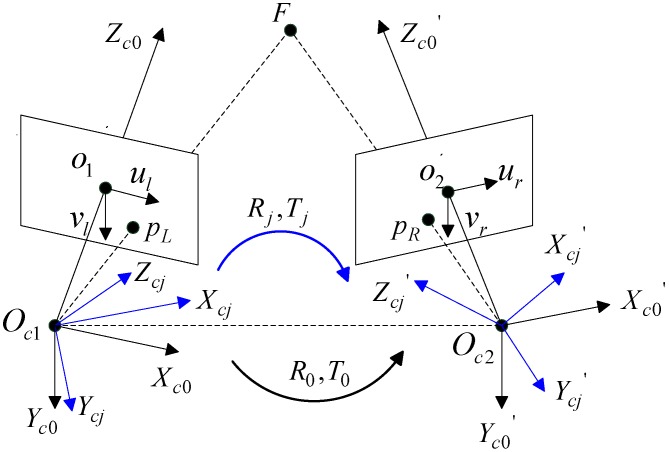
Mathematical model of binocular stereo vision.

The binocular stereo vision (BSV) system with rotating and non-zooming cameras is established, as shown in Figure 1. We describe the intrinsic parameter matrices ***K****_*1*_* and ***K****_*2*_* of the left and right camera as Equation (1), where *f_uL_*, *f_vL_* and *f_uR_*, *f_vR_* represent the focal lengths in the column and row directions for the left and right cameras, respectively, and s denotes the skewness of the two image axes, which remains zero in this study:

(1) $$K_{1}=\left[\begin{array}{ccc} f_{uL} & s & 0\\ 0 & f_{vL} & 0\\ 0 & 0 & 1 \end{array}\right],\;K_{2}=\left[\begin{array}{ccc} f_{uR} & s & 0\\ 0 & f_{vR} & 0\\ 0 & 0 & 1 \end{array}\right]\;$$

We define the pixel coordinate of the principal point of the left and right cameras’ image plane as (*u_*0*L_*, *v_*0*L_*) and (*u_*0*R_*, *v_*0*R_*), respectively. The distortion coefficient vector of a single camera is defined as ***k_c_*** = [*k_c*1*_*, *k_c*2*_*, *k_c*3*_*, *k_c*4*_*, *k_c*5*_*], where *k_c*1*_*, *k_c*2*_* and *k_c*5*_* are respectively the two, four, and six order radial distortion coefficients, and *k_c*3*_*, *k_c*4*_* are tangential distortion coefficients. If we have the distortion coordinates (*u_d_*, *v_d_*) of one point on the imaging plane, the de-distortion coordinates (*u*, *v*) of this point can be obtained according to Equations (2) and (3):

(2) $$u=f_{u}x_{n}+u_{0},\;v=f_{v}y_{n}+v_{0}\;$$

(3) $$\{\begin{array}{c} \begin{array}{l} x_{d}=\frac{u_{d}-u_{0}}{f_{u}}\\ y_{d}=\frac{v_{d}-v_{0}}{f_{v}}\\ x_{d}=x_{n}(1+k_{c1}r^{2}+k_{c2}r^{4}+k_{c5}r^{6})+2k_{c3}x_{n}y_{n}+k_{c4}(r^{2}+2{x_{n}}^{2}) \end{array}\\ \begin{array}{l} y_{d}=y_{n}(1+k_{c1}r^{2}+k_{c2}r^{4}+k_{c5}r^{6})+k_{c3}(r^{2}+2{y_{n}}^{2})+2k_{c4}x_{n}y_{n}\\ r^{2}={x_{n}}^{2}+{y_{n}}^{2} \end{array} \end{array}\;$$

where (*u_*0*_*, *v_*0*_*) is the camera’s principal point and *f_u_*, *f_v_* represent the focal lengths in the column and row directions, (*x_n_*, *y_n_*) and (*x_d_*, *y_d_*) are respectively the de-distortion coordinates and distortion coordinates on the imaging plane with normalized focal length.

The extrinsic parameters of BSV include rotation matrix ***R*** and translation vector ***T***(***T****_x_*, ***T****_y_*, ***T****_z_*). The vector ***om*** is the Rodrigues representation of the rotation matrix, and the rotation matrix can be easily obtained using the Rodrigues transformation. In this study, we use three Euler angles rotating around the X-axis, Y-axis, and Z-axis (denoted by *r_x_*, *r_y_*, and *r_z_*, respectively) to represent the rotation matrix ***R*** as shown in Equation (4). Subsequently, *r_x_*, *r_y_*, *r_z_* can be converted by matrix ***R*** according to Equation (5), where ***R_ij_*** (*i*, *j* = *1*, *2*, *3*) represents the element of matrix ***R*** in the *i*-th row and *j*-th column:

(4) $$R_{3\times 3}=R_{z}.R_{x}.R_{y}\;$$

where $R_{x}=\left[\begin{array}{ccc} 1 & 0 & 0\\ 0 & \cos(r_{x}) & \sin(r_{x})\\ 0 & -\sin(r_{x}) & \cos(r_{x}) \end{array}\right]$, $R_{y}=\left[\begin{array}{ccc} \cos(r_{y}) & 0 & -\sin(r_{y})\\ 0 & 1 & 0\\ \sin(r_{y}) & 0 & \cos(r_{y}) \end{array}\right]$, $R_{z}=\left[\begin{array}{ccc} \cos(r_{z}) & \sin(r_{z}) & 0\\ -\sin(r_{z}) & \cos(r_{z}) & 0\\ 0 & 0 & 1 \end{array}\right]$.

(5) $$r_{x}=-\tan^{-1}\frac{R_{32}}{\sqrt{{R_{31}}^{2}+{R_{33}}^{2}}},\;r_{y}=\tan^{-1}\frac{R_{31}}{R_{33}},\;r_{z}=\tan^{-1}\frac{R_{12}}{R_{22}}\;$$

The coordinate systems of the left and right cameras at the initial position are *O_c*1*_-X_c*0*_Y_c*0*_Z_c*0*_* and *O_c*2*_-X_c*0*_’Y_c*0*_’Z_c*0*_’*, respectively. The cameras are stationary but can rotate in horizontal and vertical directions. The coordinate systems of the two cameras after the *j*-th rotation are denoted by *O_c*1*_-X_cj_Y_cj_Z_cj_* and *O_c*2*_-X_cj_’Y_cj_’Z_cj_’*. We define the rotation matrix and the translation vector of the coordinate system *O_c*1*_-X_c*0*_Y_c*0*_Z_c*0*_* relative to the coordinate system *O_c*2*_-X_c*0*_’Y_c*0*_’Z_c*0*_’* as ***R****_*0*_* and ***T****_*0*_*, as shown in Equation (6). Similarly, the rotation matrix and translation vector after the *j*-th rotation is denoted by ***R****_j_* and ***T****_j_*:

(6) $$\left[\begin{array}{c} {X_{c0}}^{\prime }\\ {Y_{c0}}^{\prime }\\ {Z_{c0}}^{\prime } \end{array}\right]=R_{0}\left[\begin{array}{c} X_{c0}\\ Y_{c0}\\ Z_{c0} \end{array}\right]+T_{0}\;$$

An arbitrary point in 3D is denoted by P, which has two projection points, namely, p*_L_*(*u_L_*, *v_L_*) and p*_R_*(*u_R_*, *v_R_*), on the left and right camera image planes after the *j*-th rotation, respectively. It should be emphasized that the pixel coordinates below are de-distortion coordinates. We define the left camera coordinate system as the world coordinate system, and the origin is the optical center of the left camera. According to the geometric properties of the optical imaging, we have equations Equations (7) and (8), where *d_xL_*, *d_yL_* and *d_xR_*, *d_yR_* represent the physical dimensions of the unit pixel of the left and right cameras in the column and row directions, respectively. Given that the two cameras of the same configuration are used in this study, we define *d_xL_* = *d_xR_* = *dx*, *d_yL_* = *d_yR_* = *dy*. According to Equations (7) and (8), the 3D coordinates of *P*(*X_cj_*, *Y_cj_*, *Z_cj_*) in world coordinates can be solved by the least square method after cameras intrinsic and extrinsic parameters are obtained:

(7) $$Z_{cj}\left[\begin{array}{c} (u_{L}-u_{0L})d_{xL}\\ (v_{L}-v_{0L})d_{yL}\\ 1 \end{array}\right]=K_{1}\left[\begin{array}{c} X_{cj}\\ Y_{cj}\\ Z_{cj} \end{array}\right]\;$$

(8) $${Z_{cj}}^{\prime }\left[\begin{array}{c} (u_{R}-u_{0R})d_{xR}\\ (v_{R}-v_{0R})d_{yR}\\ 1 \end{array}\right]=K_{2}R_{j}\left[\begin{array}{c} X_{cj}\\ Y_{cj}\\ Z_{cj} \end{array}\right]+K_{2}T_{j}\;$$

### 2.2. Extrinsic Parameter Calibration Using a Single Feature Point

**Figure 2:**
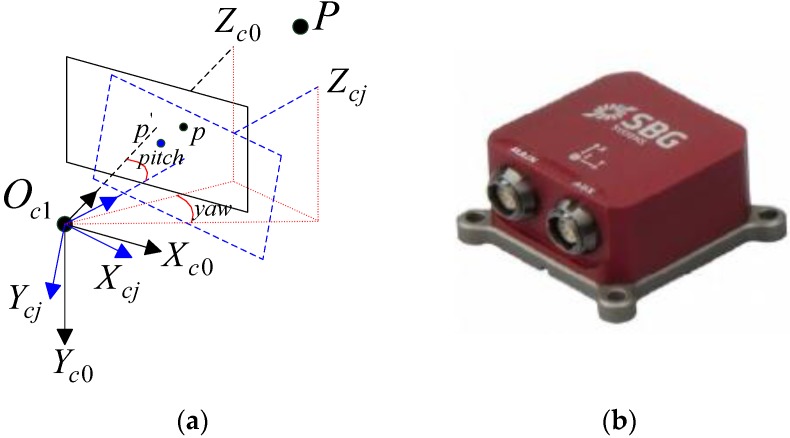
Dynamic mathematical model of the left camera and angle measuring instrument: (**a**) Rotation model of the left camera; (**b**) SBG angle measuring instrument.

This study aims to calibrate the extrinsic parameters of the BSV system when the two cameras rotate. The intrinsic parameters of the left and right cameras and the translation vector of the two cameras at the initial position can be calibrated in advance offline. As shown in Figure 2a, the coordinate system of the left camera at the initial position is *O_c*1*_-X_c*0*_Y_c*0*_Z_c*0*_*, and point *p* in the image plane is the projection point of the feature point *P* in the 3D world coordinate system at initial time. Assuming the left camera rotates to the position of the blue dotted rectangle after the *j*-th rotation, the coordinate system of the camera at this moment is *O_c*1*_-X_cj_Y_cj_Z_cj_*, and point *p’* in the image plane is the projection point of the same feature point *P* at this time. The rotation angle of the camera in horizontal and vertical directions is denoted by yaw and pitch, respectively. The approximate values of these two attitude angles can be obtained in real time using the SBG angle measuring instrument, as shown in Figure 2b, in which the precision is ±1°. Let *P*(*X, Y, Z*) represent the 3D coordinates of point *P* in *O_c*1*_-X_c*0*_Y_c*0*_Z_c*0*_*, and *p*(*u_*0*_*, *v_*0*_*) and *p’*(*u_j_*, *v_j_*) represent the image pixel coordinates in the image plane.

To simplify the notation, sin(yaw) and cos(yaw) are abbreviated as *Sy* and *Cy*, respectively. Meanwhile, sin(pitch) and cos(pitch) are abbreviated as *Sp* and *Cp*, respectively. According to the geometric imaging principle, Equations (9) and (10) are obtained for the left camera:

(9) $$Z_{c0}\left[\begin{array}{c} (u_{0}-u_{0L})dx\\ (v_{0}-v_{0L})dy\\ 1 \end{array}\right]=K\left[\begin{array}{c} X\\ Y\\ Z \end{array}\right]\;$$

(10) $$Z_{cj}\left[\begin{array}{c} (u_{j}-u_{0L})dx\\ (v_{j}-v_{0L})dy\\ 1 \end{array}\right]=KR_{j}\left[\begin{array}{c} X\\ Y\\ Z \end{array}\right]\;$$

where $K=\left[\begin{array}{ccc} \lambda f & 0 & 0\\ 0 & f & 0\\ 0 & 0 & 1 \end{array}\right]$, $R_{j}=\left[\begin{array}{ccc} Cy & 0 & -Sy\\ SySp & Cp & CySp\\ SyCp & -Sp & CyCp \end{array}\right]$, $f=f_{vL}\times dy$, $\lambda =\frac{f_{uL}}{f_{vL}}$.

Equations (9) and (10) are simply written as *Z_c*0*_*[*U_*0*_ V_*0*_ 1*]*^T^* = ***K***[*X Y Z*]*^T^* and *Z_cj_*[*U_j_ V_j_ 1*]*^T^* = ***KR_j_***[*X Y Z*]*^T^*. Thus, a mapping relationship exists between the projection points of the same single feature point in the image plane at the initial position and the image plane after the *j*-th rotation, expressed as Equation (11). This relationship is known as inter-image homography:

(11) $$\mu \left[\begin{array}{c} U_{j}\\ V_{j}\\ 1 \end{array}\right]=KR_{j}K^{-1}\left[\begin{array}{c} U_{0}\\ V_{0}\\ 1 \end{array}\right]\;$$

where *μ* is the scale factor and *μ* = *Z_cj_/Z_c*0*_*.

According to Equation (11), the following two linear equations for *Sy* and *Cy*, as shown in Equation (12), can be derived. Equation (12) can be simply represented by a matrix equation, that is, ***A****_*2*×**2**_*[*Sy Cy*]*^T^* = ***b****_*2*×**1**_*. So we can convert the imaging model into a linear model with respect to sine and cosine of the yaw, where each element in the augmented coefficient matrix is a function of the single variable pitch. Subsequently, the determinant value of the matrix ***A*** in Equation (13) can be obtained:

(12) $$\left\{ \begin{array}{l} (U_{j}U_{0}Cp+\lambda^{2}f^{2})Sy+\lambda f(U_{j}Cp-U_{0})Cy=\lambda V_{0}U_{j}Sp\\ (U_{0}V_{j}Cp-fU_{0}Sp)Sy+\lambda f(V_{j}Cp-fSp)Cy=\lambda V_{j}V_{0}Sp+\lambda fV_{0}Cp \end{array}\right.\;$$

(13) $$det(A)=\left|\begin{array}{cc} U_{j}U_{0}Cp+\lambda^{2}f^{2} & \lambda f(U_{j}Cp-U_{0})\\ \left(U_{0}V_{j}Cp-fU_{0}Sp\right) & \lambda f(V_{j}Cp-fSp) \end{array}\right|=\lambda f(V_{j}Cp-fSp)(\lambda^{2}f^{2}+{U_{0}}^{2})\;$$

If det(***A***) = 0, then *V_j_Cp*-*fSp* = 0. Thus, tan(pitch) = *V_j_/f*. In the following, the tangent value of the angle pitch is simply denoted by *Tp*. If det(***A***) ≠ 0, then the solution of *Sy* and *Cy*, expressed in Equations (14) and (15), can be obtained according to the linear equation principle:

(14) $$Sy=\frac{\left|\begin{array}{cc} \lambda V_{0}U_{j}Sp & \lambda f(U_{j}Cp-U_{0})\\ \lambda V_{j}V_{0}Sp+\lambda fV_{0}Cp & \lambda f(V_{j}Cp-fSp) \end{array}\right|}{det(A)}=\lambda^{2}fV_{0}\frac{U_{0}(V_{j}Sp+fCp)-fU_{j}}{det(A)}\;$$

(15) $$Cy=\frac{\left|\begin{array}{cc} U_{1}U_{0}Cp+\lambda^{2}f^{2} & \lambda V_{0}U_{1}Sp\\ U_{0}V_{1}Cp-fU_{0}Sp & \lambda V_{1}V_{0}Sp+\lambda fV_{0}Cp \end{array}\right|}{det(A)}=\lambda V_{0}f\frac{U_{1}U_{0}+\lambda^{2}f(V_{1}Sp+fCp)}{det(A)}\;$$

Given that *Sy^2^* + *Cy^2^* = 1, Equation (16) can be derived:

(16) $$aSp^{2}+bCp^{2}+cSpCp+d=0\;$$

where $\left\{ \begin{array}{l} a=\lambda^{2}{V_{0}}^{2}{V_{j}}^{2}-f^{2}(\lambda^{2}f^{2}+{U_{0}}^{2})\\ b=\lambda^{2}{V_{0}}^{2}f^{2}-{V_{j}}^{2}(\lambda^{2}f^{2}+{U_{0}}^{2})\\ c=2V_{j}f(\lambda^{2}{V_{0}}^{2}+\lambda^{2}f^{2}+{U_{0}}^{2})\\ d={V_{0}}^{2}{U_{j}}^{2} \end{array}\right.$.

Subsequently, a quadratic equation of variable *Tp*, expressed as Equation (17), can be derived if Equation (16) is divided by the square of *Cp*. So the linear model of the pitch and yaw are transformed into a quadratic equation of the tangent value of the pitch:

(17) $$mTp^{2}+nTp+q=0\;$$

where $\left\{ \begin{array}{l} m={V_{0}}^{2}(\lambda^{2}{V_{j}}^{2}+{U_{j}}^{2})-f^{2}(\lambda^{2}f^{2}+{U_{0}}^{2})\\ n=2V_{j}f[\lambda^{2}({V_{0}}^{2}+f^{2})+{U_{0}}^{2}]\\ q={V_{0}}^{2}({U_{j}}^{2}+\lambda^{2}f^{2})-{V_{j}}^{2}(\lambda^{2}f^{2}+{U_{0}}^{2}) \end{array}\right.$.

In the first case, if *m* = 0, then *Tp* = −*q/n*. In the second, if *m* ≠ 0, then the solution of *Tp* can be obtained using Equation (18) because the quadratic equation must contain real roots:

(18) $$Tp=\frac{-n\pm \sqrt{n^{2}-4mq}}{2m}\;$$

Notably, Equation (18) contains two solutions at the most. Hence, we utilize the SBG angular measurement instrument in this study to obtain the approximate value of the rotation angle pitch to eliminate the wrong solution. Therefore, in the two cases mentioned above, the correct solution of *Tp* can be uniquely determined. Given that the rotating angle pitch of the camera in the vertical direction satisfies −90° < pitch < 90°, we have *Cp* > 0. Subsequently, the sine and cosine values of the angle pitch, namely, *Sp* and *Cp*, can be obtained using Equation (19) according to the trigonometric function theorem:

(19) $$Cp=\sqrt{\frac{1}{1+Tp^{2}}},\;Sp=Tp\times Cp\;$$

Once the closed-form solutions of sine and cosine values of the angle pitch are obtained, the rotation angle yaw of the camera in the horizontal direction can be calculated linearly according to Equation (12), that is, [*Sy Cy*]*^T^* = ***A****^−1^**b***. Thus, the rotation angles of the left camera in two directions after the rotation can be uniquely determined. Subsequently, the rotation matrix ***R_lj_*** of the left camera relative to its initial position after the *j*-th rotation can be obtained. Similarly with the left camera, the closed-form solutions of the angle pitch and yaw of the right camera after the *j*-th rotation can also be calculated by using the same feature point with the aid of SBG system. Then the rotation matrix ***R_rj_*** of the right camera relative to its initial position after the *j*-th rotation can be obtained. The calibration method is fast and linear, thereby avoiding the problem of the local optimal solution of iterative algorithms.

In this work, the pedestal of the left and right cameras are fixed, the coordinate systems *O_c*1*_*-*X_c*0*_Y_c*0*_Z_c*0*_* and *O_c*2*_-X_c*0*_’Y_c*0*_’Z_c*0*_’* of the left and right cameras at the initial position can be mapped to the coordinate systems *O_c*1*_-X_cj_Y_cj_Z_cj_* and *O_c*2*_-X_cj_’Y_cj_’Z_cj_’*, respectively, after the *j*-th rotation according to the rotation theorem with a zero translation vector, expressed as Equation (20):

(20) $$\left[\begin{array}{c} X_{cj}\\ Y_{cj}\\ Z_{cj} \end{array}\right]=R_{lj}\left[\begin{array}{c} X_{c0}\\ Y_{c0}\\ Z_{c0} \end{array}\right],\;\left[\begin{array}{c} {X_{cj}}^{\prime }\\ {Y_{cj}}^{\prime }\\ {Z_{cj}}^{\prime } \end{array}\right]=R_{rj}\left[\begin{array}{c} {X_{c0}}^{\prime }\\ {Y_{c0}}^{\prime }\\ {Z_{c0}}^{\prime } \end{array}\right]\;$$

If the rotation matrix ***R****_*0*_* and translation vector ***T****_*0*_* of the BSV system at the initial position are known in advance, the mapping relationship between the coordinate system *O_c*1*_-X_cj_Y_cj_Z_cj_* and *O_c*2*_-X_cj_’Y_cj_’Z_cj_’*, as shown in Equation (21), can be obtained by combining Equations (6) and (20). As can be seen from Equation (21), the rotating matrix ***R****_j_* and translation vector ***T****_j_*(***T****_x_*, ***T**_y_*, ***T**_z_*) of the BSV system after the *j*-th rotation can be obtained as shown in Equation (22). The three Euler angles (i.e., *r_x_*, *r_y_*, and *r_z_*) representing matrix ***R****_j_* can be solved by Equation (5), and 3D coordinates (*X*, *Y*, *Z*) of the feature point can be estimated by Equations (7) and (8):

(21) $$\left[\begin{array}{c} {X_{cj}}^{\prime }\\ {Y_{cj}}^{\prime }\\ {Z_{cj}}^{\prime } \end{array}\right]=R_{lj}R_{0}{R_{rj}}^{-1}\left[\begin{array}{c} X_{cj}\\ Y_{cj}\\ Z_{cj} \end{array}\right]+R_{rj}T_{0}\;$$

(22) $$R_{j}=R_{lj}R_{0}{R_{rj}}^{-1},\;T_{j}={\left[\begin{array}{ccc} T_{x} & T_{y} & T_{z} \end{array}\right]}^{T}=R_{rj}T_{0}\;$$

## 3. Feasibility Analysis 

**Figure 3:**
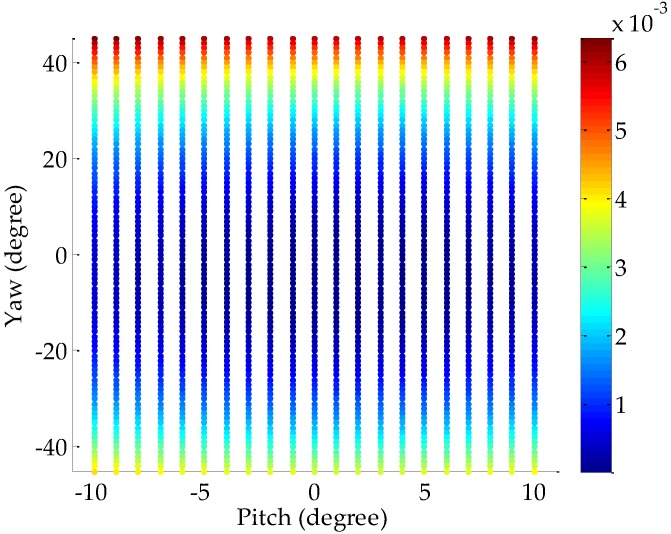
RMS versus the posture of the camera.

In this work, the left and right cameras can rotate from −45° to 45° in the horizontal direction and from −10° to 10° in the vertical direction, respectively. To investigate the performance of the proposed method with respect to the posture of one arbitrary camera, we assume that the focal length of this camera is 16 mm and the image size is 1360 pixels × 600 pixels. We vary the angle pitch from −10° to 10°, and the angle yaw from −45° to 45° with an interval of 1°. We simulate 256 pairs of matched feature points between the camera’s initial position and the position after the rotation for each posture. Hence, 256 repetitive trials are implemented and the Gaussian noise with zero mean and a standard deviation of 0.5 pixel are added to the feature points. Given that the key of the algorithm mentioned in Section 2 lies in the calculation of the angle pitch, we measure the root mean square (RMS) errors between the true values of the pitch and its estimated values to evaluate the calculation accuracy. As shown in Figure 3, the RMS increases with the increasing rotation angle but does not exceed 0.007°, implying that the proposed algorithm is suitable for all poses of the left and right cameras.

**Figure 4:**
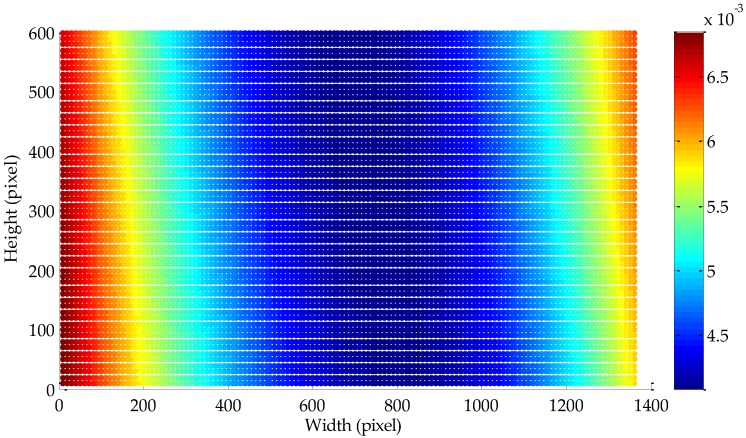
RMS versus the pixel coordinates of the feature point.

To investigate the performance with respect to the location of the pixel coordinates, we simulate a total of 8160 pixel points in the image at the initial position of the camera and simple at 10-pixel intervals. The corresponding matched pixel points after the rotation can also be simulated according to these pixel points. For each pixel coordinate location, 256 repetitive trials are implemented, and the Gaussian noise with zero mean and a standard deviation of 0.5 pixel are added to the feature points. The RMS mentioned above with respect to the pixel coordinates is shown in Figure 4. As presented in the figure, the RMS value is less than 0.007°, implying that the proposed method is feasible regardless of the pixel coordinates of the feature point.

## 4. Experiments

### 4.1. Computer Simulation

**Figure 5:**
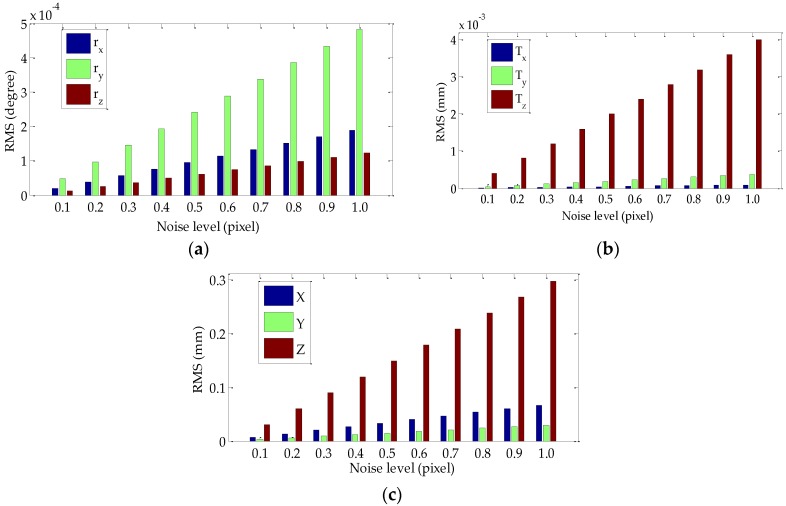
Performance of the proposed calibration method with respect to noise: (**a**) RMS of *r_x_*, *r_y_*, and *r_z_* at various noise levels; (**b**) RMS of *T_x_*, *T_y_*, and *T_z_* at various noise levels; (**c**) RMS of *X*, *Y*, and *Z* at various noise levels.

The algorithm proposed in Section 2 is a self-calibration method by using a single feature point. The extraction of the pixel coordinates of the feature points significantly affects the calibration accuracy. To evaluate the performance of the proposed method, we simulate 216 pairs of matched feature points between the initial position of binocular stereo vision and the position after the rotation. The simulation assumptions are as follows. The image size of the left and right virtual cameras are 1360 pixels × 600 pixels, and the focal length is 16 mm and 18 mm, respectively. The Rodrigues vector ***om*** and translation vector ***T_*0*_*** at the initial position is ***om*** = [−0.00281, 0.05055, 0.03414] and ***T_*0*_*** = [−304.5563, −3.8191, 27.5258]*^T^*. The Rodrigues vector ***om***’ and ***T***’ after the rotation is ***om***’ = [−0.02979, 0.13893, 0.04427] and **T**’ = [−296.8178, −0.8696, −5.7945]*^T^*. The units of the translation vector are millimeters. In each simulation experiment, a pair of feature points is randomly selected for the extrinsic parameters estimation, and the experiment is repeated 216 times in each noise level. The RMS values of the three Euler angles (i.e., *r_x_*, *r_y_*, and *r_z_*), the translation vector ***T*** = [***T****_x_*, ***T****_y_*, ***T****_z_*]*^T^*, and 3D reconstruction coordinates (*X*, *Y*, *Z*) are used to evaluate the calibration accuracy, as shown in Figure 5. The Gaussian noise with zero mean and standard deviation of 0.1–1 pixel with an interval of 0.1 pixel are added into the feature points. As shown in Figure 5, the RMS increases linearly with the increasing noise level, but the calibration error of the extrinsic parameters and the 3D reconstruction error are low when the noise level reaches 1 pixel.

### 4.2. Physical Experiment

**Figure 6:**
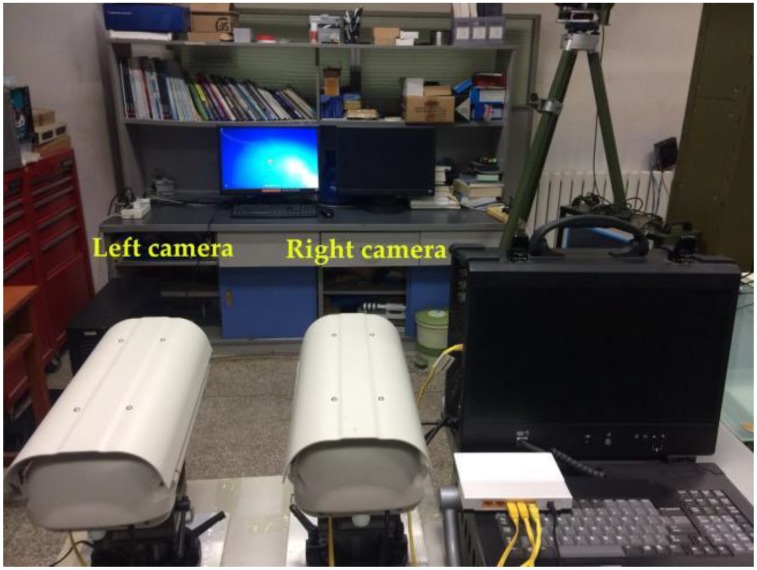
Experimental equipment of the binocular stereo vision.

To verify the proposed self-calibration method, the system of binocular stereo vision with rotating cameras is constructed, as shown in Figure 6. Two gigabit network cameras with the same configuration are installed on the horizontal platform. The two cameras can only rotate in the horizontal and vertical directions but is sufficient to adjust the field of view. The captured images are transmitted into the computer via a network cable. The image size of the left and right cameras is 1360 pixels × 600 pixels, and the physical size of the unit pixel in the column and row directions is 6.45 µm. Given that the left and right cameras are non-zooming, the intrinsic and extrinsic parameters of the cameras at the initial position can be calibrated in advance. Currently, Zhang’s checkerboard calibration method is extensively used in binocular stereo vision because of its convenience, low cost, and high precision. The intrinsic parameters, including the focal length, principal point, and distortion coefficients, of the left and right cameras obtained by using Zhang’s method are shown in Table 1, as well as the Rodrigues rotation om and translation vector *T_*0*_* at the initial position.

**Figure 7:**
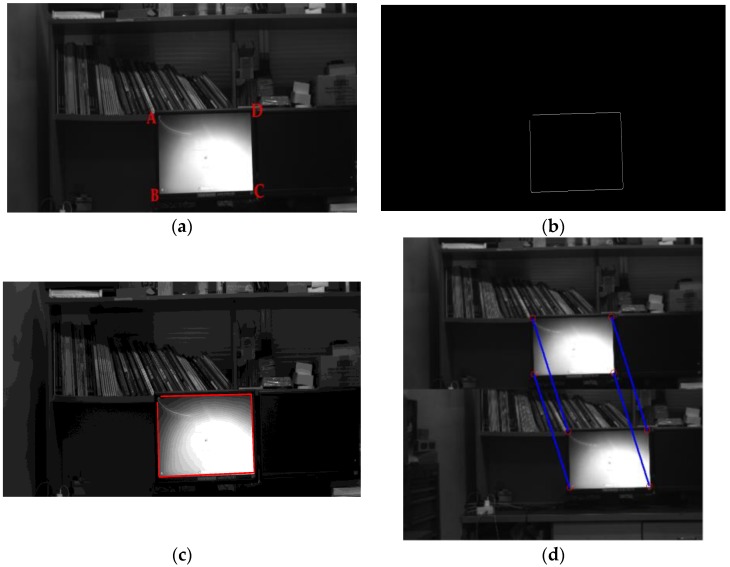
Image processing: (**a**) Source image; (**b**) Canny edge detection; (**c**) Hough line detection; (**d**) Feature point matching.

In this experiment, the top-left corner point *A* of the display screen in Figure 7a is selected as the single feature point. The remaining three feature points are used for accuracy validation. The edge of the display screen is extracted using the Canny detection algorithm, as shown in Figure 7b. The four straight lines of the display screen’s contour can be identified through the Hough line detection, as shown in Figure 7c. The intersections of these lines are the four corners of the display screen, and the pixel coordinates of the corners can be extracted to the subpixel level. The feature point matching before and after the rotation is presented in Figure 7d.

After the initial positions of the two cameras are determined, the first images of the display screen captured by the left and right cameras, are used as the reference frame. In each trial, the captured image is used as the current processing frame for the left and right camera after the cameras rotated to a certain position, and the output values of the SBG system are regarded as the approximate values of the rotation angle pitch and yaw. After the matching between the feature point in the current and reference images for the left and right cameras, respectively, is accomplished, the rotation angles of each camera in the two directions can be calculated using the proposed method in Section 2. Hence, the rotation matrix and the translation vector of the binocular stereo vision after the rotation can be obtained. In this work, Zhang’s method is also used to implement the calibration after each rotation, and the calibration results are considered to be reference values and are compared with our data.

**Figure 8:**
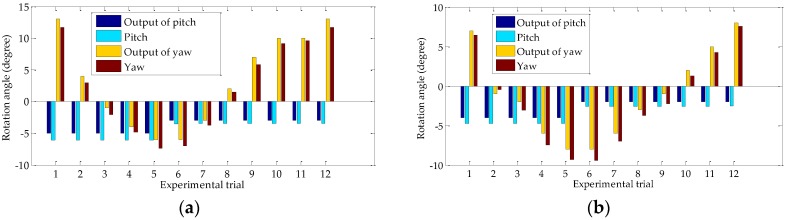
Our calculated rotation angles and the corresponding rough values: (**a**) Rotation angles of the left camera; (**b**) Rotation angles of the right camera.

This experiment is repeated 12 times. Figure 8a presents the rotation angles of the left camera in the two directions obtained by using proposed method and the corresponding approximate values. Figure 8b presents that of the right camera. The angles in Figure 8 based on our calculations are close to the approximate values, thereby verifying the validity of the algorithm.

**Figure 9:**
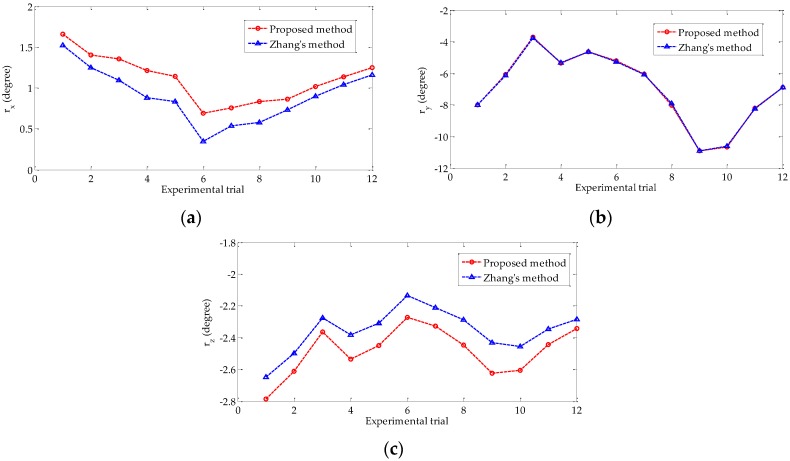
Euler angles estimated by using the proposed and Zhang’s methods: (**a**) *r_x_* of the two methods; (**b**) *r_y_* of the two methods; (**c**) *r_z_* of the two methods.

**Figure 10:**
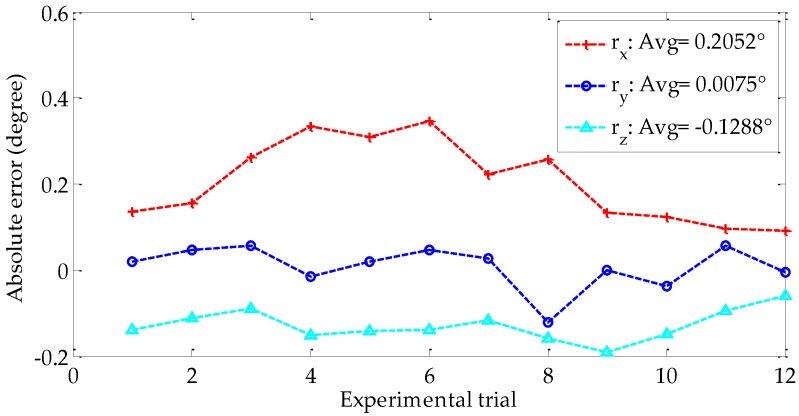
Absolute error of the three Euler angles.

Figure 9 shows the three Euler angles representing the rotation matrix, namely, *r_x_*, *r_y_*, and *r_z_*, obtained by using the proposed and Zhang’s methods in each experimental trial. As shown in Figure 9, the trend of the three angles in proposed method is the same as that of Zhang’s method. The absolute errors of the three Euler angles relative to the reference values are presented in Figure 10. The three averages of the absolute errors are less than 0.21°, and the average error of *r_x_* is the largest.

**Figure 11:**
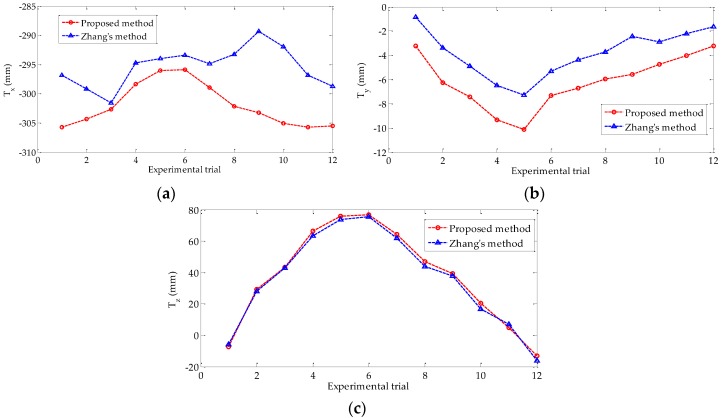
Translation vector estimated by using the proposed and Zhang’s methods: (**a**) *T_x_* of the two methods; (**b**) *T_y_* of the two methods; (**c**) *T_z_* of the two methods.

**Figure 12:**
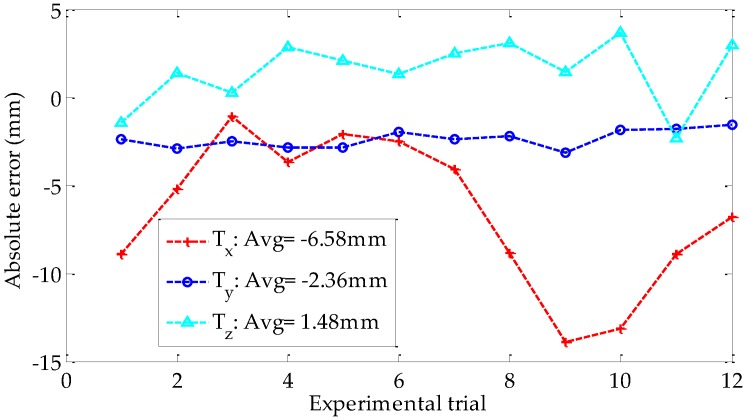
Absolute error of the translation vector.

Figure 11 shows the translation vector, namely, *T_x_*, *T_y_*, and *T_z_*, obtained by using the proposed method and Zhang’s methods in each experimental trial. Undoubtedly, the calibration accuracy of Zhang’s method must be much higher than that of our self-calibration method by using a single feature point. However, the calibration results estimated by using the proposed method are close to those of Zhang’s method, which proves the reliability of the proposed method. The absolute errors of the translation vector relative to the reference values are presented in Figure 12. The three averages of the absolute errors are less than 6.6 mm, and the average error of *T_x_* is the largest.

**Figure 13:**
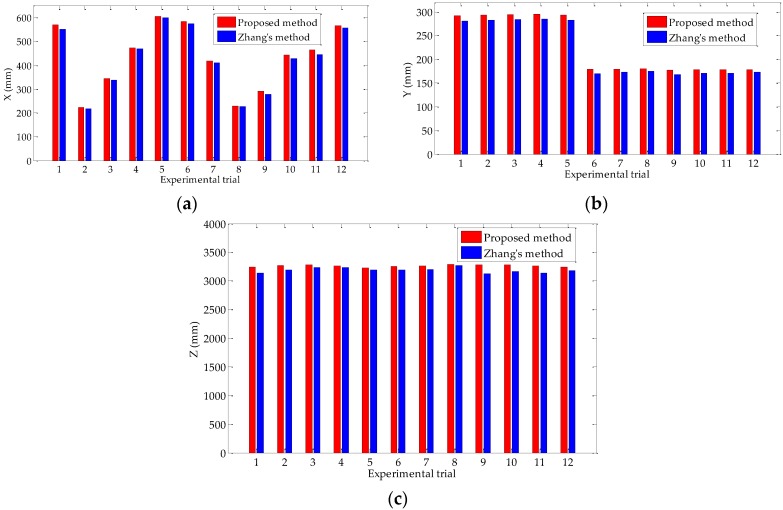
The 3D coordinates estimated by using the proposed method and Zhang’s method: (**a**) X of the two methods; (**b**) Y of the two methods; (**c**) Z of the two methods.

**Figure 14:**
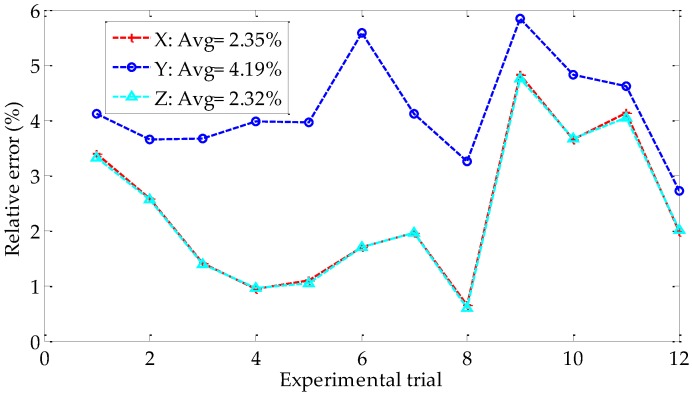
The relative error of the 3D coordinates.

In each trial, we calculate the 3D coordinates of the feature points B, C, and D on the display screen after the rotation, as shown in Figure 7a. The averages of the 3D coordinates are used for the evaluation of calibration accuracy. As shown in Figure 13, we compare the data obtained by using the proposed method with that calculated by using Zhang’s method. The figure shows that the 3D reconstruction coordinates of the proposed method is close to that of Zhang’s method, which proves the validity of the proposed method. The relative errors of the 3D coordinates with respect to the reference values are presented in Figure 14. The averages of the relative errors are less than 4.2%, and the relative error on Y-axis is the largest. The proposed method is suitable for the occasions where high accuracy of 3D reconstruction is not required.

## 5. Conclusions

In this study, we present a novel self-calibration method for extrinsic parameter estimation of a rotating binocular stereo vision by using a single feature point. This is achieved by assuming the intrinsic parameters of the left and right cameras are known in advance, as well as the rotation matrix and the translation vector at the initial position. Only the rotation angles of the left and right cameras in the vertical and horizontal directions, that is, pitch and yaw, must be calculated after the rotation. We transformed the geometric imaging model of the pitch and yaw into a quadratic equation of the tangent value of the pitch. The closed-form solutions of the pitch and yaw are obtained with the aid of SBG equipment. Once the pitch and yaw are uniquely determined, the rotation matrix of the BSV system and the three Euler angles representing rotation matrix can be calculated according to rotation theorem. The translation vector are estimated by the rotation matrix of the right camera and the initial translation vector.

The proposed method is non-iterative, thus addressing the problem of not obtaining a global optimal solution in iterative algorithms. Under extreme conditions, we limit the number of feature points for calibration to a minimum, remarkably shortening the consumption time of the feature point matching and allowing the possibility to calibrate the extrinsic parameters of the rotating binocular stereo vision in real time. Computer simulations prove the feasibility of the proposed method, and the error of the extrinsic parameters and 3D coordinate reconstruction are minimal when the noise level was high. To validate the feasibility of the proposed method, we compare it with Zhang’s method. Although Zhang’s method exhibits a much higher precision, our calibration results from repeated experiments are close to the reference values estimated by Zhang’s method. The primary contribution of this study is that the proposed method can be used for real time 3D coordinate estimation of dynamic binocular stereo vision in when an extremely high calibration accuracy is not required. In our future work, a real-time and highly accurate method will be simultaneously investigated.

## Figures and Tables

**Table 1:** Cameras parameters.

Camera	***f_u_*/pixels**	***f_v_*/pixels**	***u_*0*_*/pixels**	***v_*0*_*/pixels**	***k_c_***
Left	2493.09	2493.92	725.77	393.03	[−0.17, 0.18, 0.003, 0.0004, 0.00]
Right	2811.56	2811.54	692.04	371.96	[−0.13, 0.16, 0.001, 0.0003, 0.00]
***om***	[0.01085, 0.05695, 0.03387]				
***T_*0*_***/mm	[−306.9049, −4.3956, 39.6172]				
